# 

**Published:** 1907-07-15

**Authors:** 


### ANNOUNCEMENTS.

DEATH OF MRS. BODECKER.—The many friends of Dr. C. F. W. Bodecker, who for many years was a distinguished practitioner and scientific investigator of New York City, and who has resided for the last few years in Berlin, Germany, will be pained to learn that Mrs. Bodecker died at her home in Berlin, June 7th, 1907. Her death was the result of a fall which she had several months ago and which caused a spinal injury which incapacitated her for walking or other exercise. The hearty sympathy of a large number of acquaintances and friends will be with Dr. Bodecker and his two sons in their great bereavement.

CORRECTION.——Our printers in making up the June issue published an abstract of a paper by Dr. Locke as an original article, when it should have been included under the report of the procedings of the Ohio State Dental Society, and credited to *The Dental Summary*, through the courtesy of whose editor we were able to present the abstract report.

THE INDIANA DENTAL ASSOCIATION has begun active work in preparing for a Semi-Centenial Jubilee meeting to be held in Indianapolis, June 4, 5, 6 1908, celebrating the Fiftieth Anniversary of the State Association.

D. A. HOUSE, *Sec.*

ACCOMMODATIONS FOR GUESTS AT THE JAMESTOWN CONVENTION.—In view of the statements that have gone out in relation to the lack of accommodation and high hotel charges in the vicinity of the Jamestown Exposition, we, the Virginia members of the Organization Committee of the Jamestown Dental Convention, deem it wise to make a formal statement of the situation, that the members of the profession who contemplate attending the Convention at the Jamestown Exposition, Norfolk, Virginia, September 10-12, may not be mislead or deterred in their purpose:

While at this time the Exposition is not entirely completed, it presents a very attractive appearance and is well worth seeing, and by the date set for the convention, it will not only be fully completed, but will be at the very height of perfection.

Aside from any attraction that is offered by the Exposition, the assembling in Hampton Roads of the fleets of the nations of the world is amply worth the trip to the Jamestown Exposition.

The Jamestown Exposition Grounds are not located in any one city, but are nearly equi-distant from the Tidewater Cities of Norfolk, Portsmouth, Newport News, Hampton and Old Point Comfort and within thirty minutes ride by rail or water of any one of them.

The hotels and cottages of the summer resorts of Pine Beach, Luckroe Beach, Ocean View, Willoughby Beach, Cape Henry and Virginia Beach, will be utilized for the accommodation of visitors.

The suburbs of Norfolk, Portsmouth and Newport News, such as West Norfolk, Port Norfolk, South Norfolk, Berkley, Riverside, River View, Edgewater, Lamberts Point, Parke Place, have many comfortable homes that will be opened to receive guests, and are all well connected with electric lines of street railway to points of departure for the Exposition Grounds.

We append a list of some of the leading hotels and their rates per day. In order to secure these rates it will be necessary to make reservations not later than August 15th.

The Inside Inn, with a capacity of 3,000 persons, will be the official headquarters of the convention. The following are the rates of the Inside Inn.

European Plan, without bath, two persons in a room, which includes breakfast, priveleges of the Inn, and admission to the Grounds after the guest has registered at the hotel, $2.50 per day, for each person. If room is occupied by only one person, the rate will be $3.50 per day.

American Plan, without bath, two persons in a room, which includes breakfast, privileges of the Inn, luncheon, and our $1.00 evening Table D'Hote dinner with wine, admission to the grounds, after the guest has registered at the hotel, $3.50 per day for each person. If room is occupied by only one person, the rate is $4.50.

The rates for rooms fronting the sea, or the sea and pine grove:

American Plan, if room is occupied by only one person, the rate will be $6.00 per day. If room is occupied by two persons the rate will be $8.00 for the two persons.

American Plan, with bath and toilet, $8.00 per day for one person in a room, if room is occupied by two persons the rate will be $10.00 for two persons.

There are about twenty hotels in Norfolk and Portsmouth accomodating from 25 to 100 guests each, with rates at from one dollar and upward per day according to location and conveniences.

In addition to the hotels named above, there are hundreds of private boarding houses and rooming houses, at which visitors may secure accomadations at reasonable rates.

We will thank you very much to give the above information as wide publicity as you can through your esteemed journal.

Yours very truly,

R. H. Walker, Norfolk, Va.
F. W. Stiff, Richmond, Va.
H. W. Campbell, Sec'y, Organization Committee.

Norfolk, Va.
June 24th, 1907.

